# Clinical observation on healing of tarsal plate defect after reconstruction with xenogeneic acellular dermal matrix

**DOI:** 10.1186/s12886-022-02540-6

**Published:** 2022-07-29

**Authors:** Qin Huang, Yangbin Fang, Yaohua Wang, Hongfei Liao

**Affiliations:** Affiliated Eye Hospital of Nanchang University, Jiangxi Research Institute of Ophthalmology and Visual Science, Jiangxi Provincial Key Laboratory for Ophthalmology, Nanchang, Jiangxi China

**Keywords:** Xenogeneic acellular dermal matrix, Malignancy, Reconstruction

## Abstract

**Objective:**

To evaluate the safety, function, and cosmetic outcome of eyelid reconstruction using a xenogeneic acellular dermal matrix as a tarsal plate replacement in the repair of 50 to 100% eyelid defects following excision of large malignant tumours.

**Methods:**

A retrospective, non-comparative, interventional study of 21 eyes was performed over 26 months. Fourteen patients were female and seven were male. In all cases, a xenogeneic acellular dermal matrix was used for total or subtotal replacement of the tarsal plate. The central vertical height of the palpebral fissure was measured immediately after eyelid margin incision and at 1 and 6 months postoperatively.

**Results:**

In patients who underwent surgery, the mean palpebral fissure height (PFH) was not significantly different between immediately and 1 month after incision (8.10 ± 0.562 mm vs 8.17 ± 0.577 mm, respectively; *P* > 0.05). After 6 months, PFH was 8.26 ± 0.605 mm, which was significantly different from that immediately after incision (*P* < 0.05). After 6 months of follow-up, all patients had a good aesthetic appearance after eyelid reconstruction, with no obvious graft dissolution or rejection, normal eyelid activity, and normal opening, closing, and lifting function. None of the 21 patients experienced tumour recurrence during postoperative follow-up.

**Conclusion:**

The xenogeneic acellular dermal matrix was a successful tarsal plate replacement. This material is readily available, and a second surgical site is avoided. The xenogeneic acellular dermal matrix is considered a promising alternative material for tarsal replacement in future generations.

## Introduction

Eyelid resection of malignant tumours often results in eyelid loss. Although small defects can be reconstructed using direct closure, larger defects after the removal of large malignant tumours require more extensive surgery. An oculoplastic surgeon faces a major challenge in restoring eyelid anatomy and function while maintaining satisfactory cosmetic outcomes. Xenogeneic acellular dermal matrix (Xneo-ADM) has been a new material in the field of plastic surgery in recent years. The application of new tissue engineering technology removes the cellular components of the epidermis and dermis from fresh skin specimens. Antigenicity is low, and the main component is collagen fibres [1]. The tissue structure is similar to that of allogeneic sclera [2], and it has a wide range of sources, is safe and convenient to obtain, is easy to store, and has a low cost. This has gradually become a new hot spot in research on ocular plastic repair materials. Since March 2019, a retrospective, non-comparative, interventional study of 21 eyes was performed over 26 months. Twenty-one patients with moderate and severe eyelid defects after eyelid malignant tumour resection (confirmed by surgery and pathology) were treated with xenogeneic acellular dermal matrix transplantation to replace the tarsal plate simultaneously. This study aimed to determine the efficacy of the xenogeneic acellular dermal matrix as a tarsal replacement for repairing eyelid defects created after the removal of large malignant tumours, evaluate the safety of using a xenogeneic acellular dermal matrix as an alternative to tarsal plate replacement and lid reconstruction, and appraise the recurrence of the disease along with the functional and cosmetic outcomes.

### Subjects

Twenty-one patients (7 male, 14 female; 55–65 [average 68.48] years of age) who underwent eyelid reconstruction and eyelid malignant tumour resection simultaneously between March 2019 and May 2021 were selected. Of 21 cases (21 eyes), 13 (61.9%) and 8 (38.1%) involved the right and left eyes, respectively; 15 cases involved the upper eyelid, 13 cases the lower eyelid, and 7 cases both the upper and lower eyelids. Eight (38.1%) cases had eyelid defects involving more than 2/3 of the eyelid length and 13 (61.9%) cases had defects involving 1/2–2/3 of the eyelid length after tumour resection. Pathological diagnoses included eyelid adenocarcinoma in 8 cases, basal cell carcinoma in 8, eyelid squamous cell carcinoma in 3, eyelid malignant melanoma in 1, and squamous cell papilloma in 1.

The exclusion criteria were eyelid tumours with intra-orbital extension, local lymph node involvement, distant metastasis, and cases with corneal anaesthesia or gross corneal involvement or infiltration.

## Methods

Institutional ethics committee clearance was obtained with reference. Informed written consent was obtained from each patient recruited for the study in accordance with the Declaration of Helsinki. In all cases, the heterogeneous (bovine) acellular dermal matrix was obtained from a commercially available Haifu Skin Repair Membrane produced by Yantai Zhenghai Biotechnology Co., Ltd., as the implant, and the size was 20 mm × 30 mm or 30 mm × 40 mm.

### Surgical procedure

**Fig. 1:**
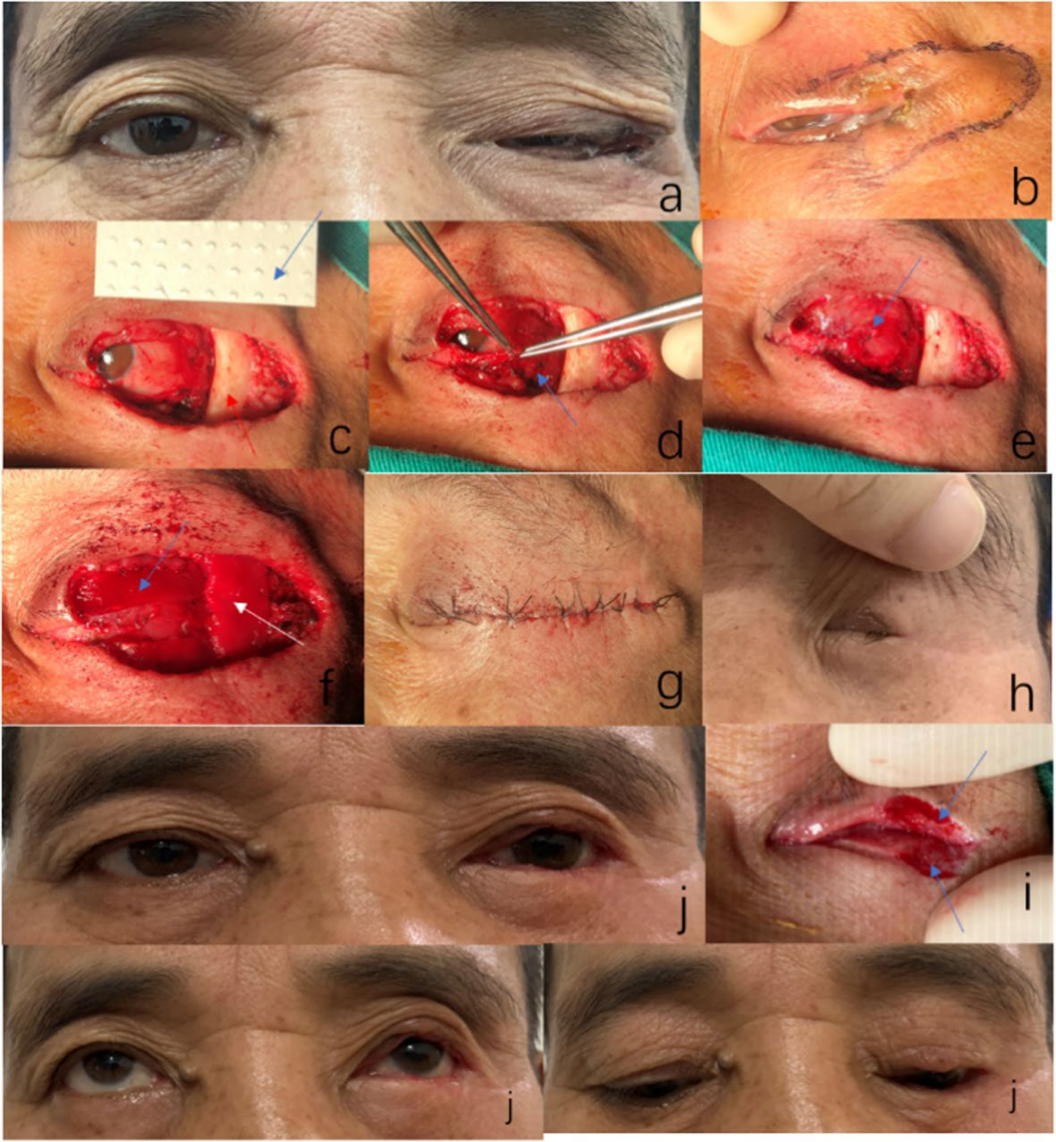
**a** A patient with basal cell carcinoma of the left eye, male, 57 years of age. **b** Skin marking of the margin beyond 3–4 mm around the mass. **c** The upper and lower lids and the temporal periosteum of the affected eye have tumor involvement. The eyelid defect (red arrow) is approximately 2/3. It is planned to use x-ADM (blue arrow) is pfor eyelid reconstruction. **d** The remaining conjunctiva and capsulopalpebral fascia (blue arrow) are detached from the upper and lower fornix. **e** Interrupted sutures are performed using 7–0 vicryl absorbable sutures to form the posterior lamina (blue arrow). **f** The conjunctiva of the upper and lower eyelid fornix is separated and sutured in the opposite position. Anatomical reduction of the eyelid plate (blue arrow) was replaced by x-ADM. The temporal periosteum was wrapped with x-ADM (white arrow). **g** The anterior lamina is made of local advancement skin flap, and eyelid margin adhesion is required. **h** Six months after eyelid reconstruction. **i** Eyelid margin incision is performed 6 months after eyelid reconstruction, and the graft is pink (blue arrow), which is integrated with the surrounding tissues. **j** The eyelids are well-shaped and open and close normally 6 months postoperatively

**Fig. 2:**
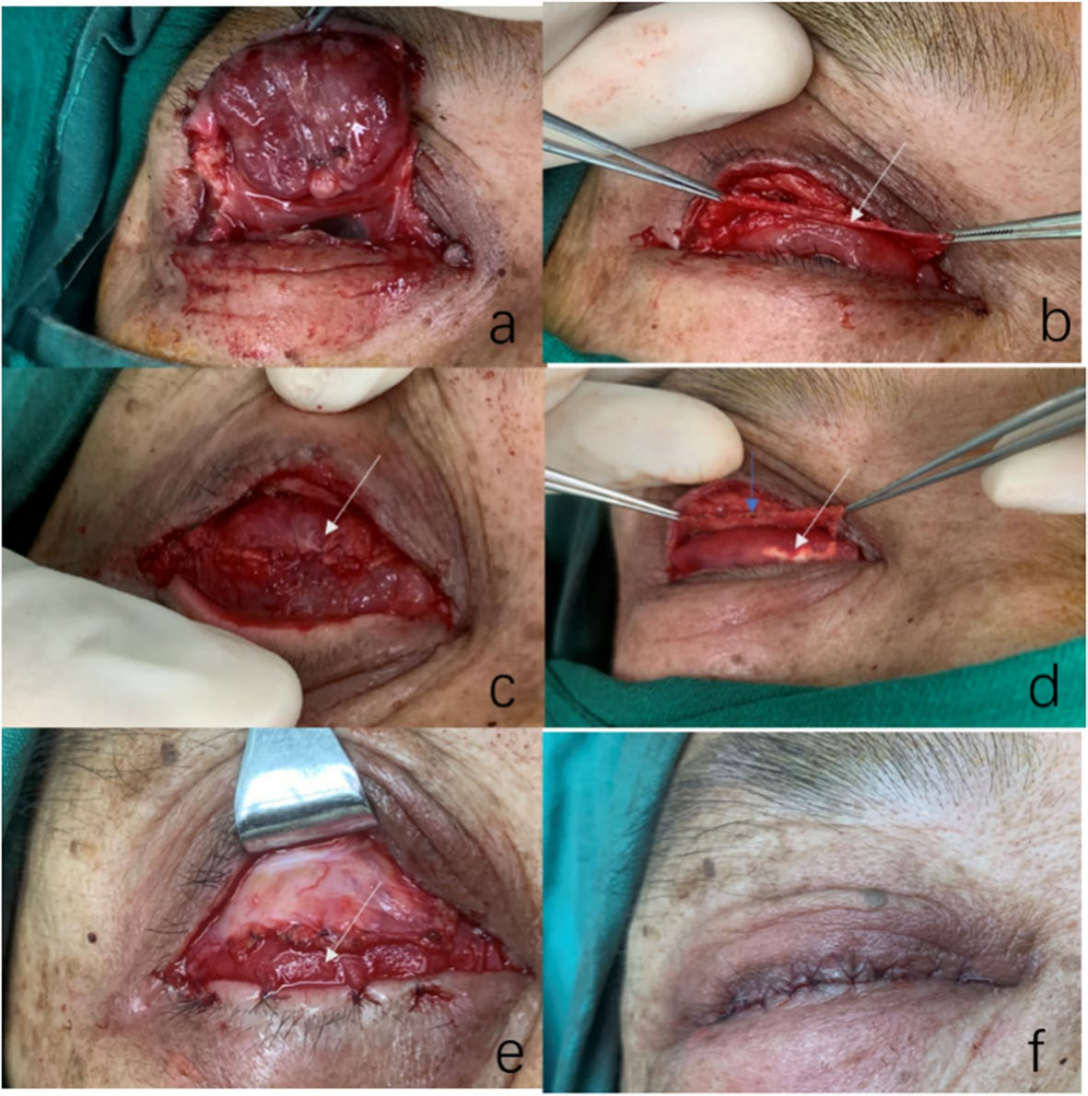
**a** A 67-year-old female patient with adenocarcinoma of the meibomian of the right eye. The eyelid defect involves the entire upper eyelid. **b** The remaining conjunctiva and capsulopalpebral fascia (white arrow) are detached from the upper fornix. **c** The Hughes flap was created in the lower eyelid conjunctiva, preserving a 4-mm conjunctiva margin to maintain eyelid function, and sutured interrupted with 7–0 vicryl to form the posterior lamina (white arrow). **d** The upper tarsal plate is replaced with Xneo -ADM (white arrow) and is separated from the residual of the levator palpebrae superioris (LPS) muscle and the orbital septum (blue arrow). **e** Xneo-ADM (white arrow) is introduced between the anterior and posterior laminae. **f** Immediately after eyelid reconstruction

In this study,all surgical procedures were performed by a single experienced surgeon with the same settings under general anaesthesia. The incision line was marked beyond 3–4 mm the healthy skin margin around the mass used methylene blue (Fig. 1b). After complete resection of the tumor, we made an incision with a scalpel along the outlined margin, beveled at a 30 angle, and the tissue was then sharply excised to an appropriate depth along a flat horizontal plan. Each peripheral margin were marked and sent to frozen section biopsy,frozen section biopsy was mandatory to exclude tumour-free margins and prevent postoperative recurrence. Next, the xenogeneic acellular dermal matrix was trimmed according to the size and shape of the tarsal defect (Fig. 1c). The remaining conjunctiva and capsulopalpebral fascia were detached from the upper and/or lower fornix (Figs. 1d and 2b) and sutured interrupted with 7–0 vicryl absorbable sutures to form the posterior lamina (Figs. 1e and 2c). The implant was then positioned over the posterior lamella and fixed with 6–0 vicryl sutures all around (Figs. 1f and 2d), the posterior lamina more in length than the implant about 0.5 mm so that the margin can be covered by a smooth conjunctival surface, pay attention to the knot faces away from the corneal surface, which can avoid corneal damage. Both ends were sutured interrupted on the residual tarsal plate or the canthal ligaments with 6–0 vicryl. During this procedure, no fluid was attached to the implant prior to pruning. The thickness of the graft was 0.7 mm in the freeze-dried state and could expand to 1 mm after water absorption. Their lengths and widths were the same. Therefore, it was not necessary to deliberately expand and reduce artificial errors during pruning. If the defect is in the upper eyelid, the implant must be tightly sutured with the residual of the levator palpebrae superioris muscle and orbital septum (Fig. 2e). If the defect is in the lower eyelid, the implant must be fixed with the orbicularis oculi muscle to ensure the normal opening and closing function of the eyelids. Because the lower eyelid plate played a major supporting role, if the defect length was greater than 2/3, the implant could be folded and thickened, which was conducive to the shaping of the lower eyelid and reduction of the risk of eyelid retraction. At the same time, it can resist partial degradation of the implant material before non-vascularisation. Single-layer implants are generally used in surgery to avoid mechanical blepharoptosis of the upper eyelid. As a scaffold, there is no need to distinguish between upper or basal surfaces. When sutured, anatomical reduction was performed as far as possible according to the anatomical position of the eyelid plate. If there is a missing part of the periosteum due to tumour involvement, the implant can also be wrapped to replace the missing periosteum (Fig. 1f). The anterior lamina, consisting of a skin muscle flap, a pedicled flap, or local advancement of the skin flap around the eyes, was the common method. Free flap transplantation is not recommended because of the insufficient blood supply. Because the reconstruction scope was large, eyelid margin adhesion was required to counter the eyelid skin contractures (Figs. 1g and 2f). All patients needed to be pressurised and bandaged for 2–3 days after the operation, which was beneficial to the close contact between the implant and the surrounding tissues. Finally, after at least 6 months, the margin of the eyelid was incised and trimmed (Fig. 1h). The eyelid margin was covered by cell migration. The implant merged with the surrounding tissue (Fig. 1i), and the defect area was completely repaired.

The patients were followed up for 6 months. The evaluation parameters of the central vertical height of palpebral fissure (PFH) were measured both immediately after eyelid margin incision and at 1 month and 6 months postoperatively; postoperative entropion, ectropion, any lid shrinkage, lagophthalmos, lid thickening, and lid margin irregularity were also measured. In each case, a corneal examination was mandatory to exclude suture-related or margin-related complications. The statistical data analysis was conducted using IBM SPSS statistics, Version 23.0. Descriptive statistical analyses were performed to calculate the means and standard deviations. The means were compared using the paired t-test, and *P* < 0.05 was considered statistically significant.

## Results

The basic patient information is presented in Table 1. The central vertical height of the palpebral fissure ranged from 7 to 9 mm (mean = 8.10 ± 0.562 mm) at the immediate incision. PFH was 8.17 ± 0.577 mm and 8.26 ± 0.605 mm at 1 month and 6 months after incision, respectively (Table 2). There was no statistically significant difference in PFH between the immediate incision and 1 month after incision or between 1 month and 6 months after incision (*P* > 0.05). There was a statistically significant difference in PFH between the immediate incision and 6 months after incision (*P* < 0.05). During follow-up, only two patients (9.5%) developed lid entropion and a large palpebral fissure accompanied by eye irritation, and both patients received radiotherapy after resection surgery, while the other cases of PFH from immediate postoperative to 6 months postoperatively were not significantly changed. Initially, all patients had mild margin irregularities, but no corneal complications were noted. Transient lagophthalmos occurred at 1 month postoperatively in 3 cases (14.2%) who were advised to blink frequently, but not overstrain. Lagophthalmos resolved 6 months postoperatively (Table 2). The cosmetic result was satisfactory in all cases, eyelid movement was good, and opening, closing, and lifting functions were normal (Fig. 1j). All 21 patients had no tumour recurrence during the postoperative follow-up.

**Table 1:** Basic patient information

Disease	Male	Female	Left eye	Right eye	Upper eyelid	Lower eyelid	Upper and lower eyelids	1/2–2/3	> 2/3
Meibomian									
adenocarcinoma	2	6	2	6	6	5	3	5	3
Basal cell carcinoma	5	3	4	4	5	6	3	6	2
Squamous cell carcinoma	0	3	1	2	2	1	0	2	1
Malignant melanoma	0	1	1	0	1	0	0	0	1
Squamous cell papilloma	0	1	0	1	1	1	1	0	1

**Table 2:** Comparison of Immediate incision parameters with postoperative parameters among the study population (*n* = 21)

Evaluation Parameter	Immediate incision mean with standard deviation	1 month after incision mean with standard deviation	6 months after incision Mean with standard deviation
Height of palpebral fissure (PFH)	8.10 ± 0.562 *	8.17 ± 0.577 **	8.26 ± 0.605 ***
Entropion upper lid	nill	nill	**2**
Ectropion lower lid	nill	nill	nill
Lagophthalmos	nill	**3**	nill
Unusual Hypertrophy	nill	nill	nill
Relapse	nill	nill	nill

1 month postoperative control immediate incision (p1), 6 months after incision control 1 month after incision.

control (p2), 6 months after incision control control immediate incision (p3)

*p1 = 0.083,**p2 = 0.104, ***p3 = 0.049

## Discussions

Surgical resection using the Mohs method is currently the main method for the treatment of malignant eyelid tumours [3]. However, large-scale full-thickness eyelid defects seriously affect the appearance of the eyelid and the protective function of the eyeball, thereby requiring eyelid reconstruction and excessive surgical resection. This scope introduces great difficulties for repair. As the scaffold tissue of the eyelid, the eyelid plate plays an important role in maintaining the eyelid shape and protecting the eyeball. Insufficient and complete deletion of the eyelid plate caused by surgical resection of malignant eyelid tumours has always been a difficult problem in eye plastic surgery (Figs. 1a and 2a). The focus of repair is on the repair of eyelid tissue. An ideal palpebral replacement material should have the following properties: good biocompatibility, good operability, easy to obtain, compatible mechanical properties, and suitable thickness and surface properties.

Current clinical applications of eyelid reconstruction and tarsal replacement materials mainly include autologous tissue materials, allogeneic tissue materials, and artificial synthetic materials. The synthetic material high-density porous polyethylene gasket (Medpor) has sufficient strength to support the eyelid and plasticity. They can be cut and shaped according to the shape of the eyelid. It has a high degree of acceptance for eyelid height and stability. However, the hardness of the Medpor gasket is obviously higher than that of the natural eyelid, the elasticity is poor, the eyelid is rigid, the surface is rough, and it is easily exposed after operation [4]. The autologous tarsal plate-conjunctival flap can provide sufficient mechanical support and a smooth conjunctival surface with good healing and a low necrosis rate. The autologous hard palate mucosa is similar in hardness and thickness to the tarsal plate and has a mucosal surface, which makes it a good replacement for tarsal plate materials, with no risk of rejection, but there will be more persistent sticky secretions in the eyes [5]. Commonly used autologous cartilage tissues are ear cartilage and nasal septal cartilage mucosa [6]. The harvest of ear cartilage is simple and its morbidity is minimal. Moreover, ear cartilage has a spherical surface and fits well to a bulbar surface [7], but it requires the surgeon to have nose surgery experience, and the operation time is long. Allogeneic sclera has become mature in clinical applications. It exhibits moderate rigidity, elasticity, and good histocompatibility. However, as human tissue, like autologous tissue, is subject to source restrictions and high cost, it cannot be mass-produced to meet the needs of a large number of clinical plastic surgery cases for implant materials. Human scleral tissue is not available in hospitals without eye bank facilities and requires certain conditions for preservation. Xenogeneic acellular dermal matrix as an emerging wound repair material has gradually been widely used in clinical practice, from the initial wound coverage of burn patients [8] to tympanic membrane repair [9], lip augmentation [10], and plastic surgery [11, 12]. It has gradually expanded to include ocular plastic surgery [13]. In this study, xenogeneic acellular dermal matrix was used as a substitute for the eyelid plate for 21 cases of eyelid malignant tumour resection and stage I eyelid reconstruction. The patients were followed up for 6 months to observe their clinical efficacy.

The acellular dermal matrix (ADM) is a new type of tissue engineering material. Studies have used acellular human dermal matrix (HADM) grafts in ophthalmic plastic and reconstructive surgery and were satisfactory at the 6-month follow-up examination [14]. In an experimental study on eyelid reconstruction with acellular xenogeneic pig dermal matrix (Xeno-ADM), eyelids implanted with Xeno-ADM had fewer inflammatory reactions, fewer infiltrating lymphocytes, and higher vascularisation with faster ingrowth of new collagen fibres, which also indicated that Xeno-ADM had good compatibility [15, 16]. Compared with the HADM and Xeno-ADM groups, no significant differences in adhesion, inflammation, fibrous tissue, or neovascularisation were noted [17], but Xeno-ADM can avoid the risk of a second surgical incision and is more ethically accepted than allogeneic ADM (human cadavers). In addition, Xeno-ADM is convenient for obtaining materials and has many sources. It can be processed into various thicknesses (1–4 mm) to meet various requirements. Therefore, the clinical application of Xeno-ADM has become increasingly extensive [18]. Some research [19] compared bovine (BADM) and porcine (PADM) cellular dermal matrices; BADM is inherently stronger than PADM at equivalent thicknesses and considerably stronger at increased thicknesses, significantly higher rates of skin erythema, and a trend towards higher complication rates (including implant loss, acellular dermal matrix loss, and reoperation rates) with PADM [20, 21]. Therefore, the implant material used in this study was derived from freeze-dried bovine products to speed up the formation of graft vascularisation as much as possible and avoid the risk of possible implant degradation [21]..

The xenogeneic dermis is specially processed using tissue engineering technology, and only the non-antigenic extracellular matrix and porous three-dimensional membrane scaffold structure and components are retained. Its spatial network structure is conducive to cell proliferation and capillary hyperplasia, and experiments have shown that this material can support epithelial growth on its surface, thereby accelerating the body’s healing process [22]. The graft is not absorbed or wrapped, but induces the formation of connective tissue in the recipient during vascularisation and eventually becomes part of the recipient’s body [23]. We encountered cases in which the eyelid margin was cut 6 months after the graft was implanted. It can be seen that the graft is closely fused with the surrounding tissues, is pink, has been completely accepted and vascularised by the recipient, and has a certain degree of hardness (Fig. 1i).

In addition, the epidermal cells and skin fibre cells that mainly cause immune responses in the skin are removed during production, effectively reducing antigenicity, inflammatory reactions, and immune rejection. Therefore, the inflammatory response induced by implantation in the body is much lower [2, 15, 16]. In this study, 21 patients were followed up for 6 months after incision. All patients with eye grafts survived, and there was no graft dissolution or rejection, indicating that this type of artificial material has good tissue compatibility.

Xeno-ADM has good mechanical properties and sufficient rigidity to support the shape of the eyelid, acts as a spacer graft in surgery to treat eyelid retraction in patients with thyroid-associated ophthalmopathy and has good functional and aesthetic improvements [24]. In our study, the range of eyelid defect area was more than 1/2, and both the upper and lower eyelids were involved in 7 cases, which cannot be reconstructed by traditional surgical methods. All the cases followded-up at 6 months after eyelid reconstruction showed good shape, good mobility, open and closed function, and normal lifting function (Fig. 1j). Although the literature [25] mentioned that the implant had the risk of retraction, we did not directly observe implant retraction in our study because the implant was already wrapped. Only three patients developed transient lagophthalmos at 1 month postoperatively, which resolved after 6 months postoperatively, although there was a statistically significant difference in PFH between the immediate postoperative period and 6 months postoperatively (*P* < 0.05). We think this is due to two patients receiving radiotherapy after eyelid reconstruction, which resulted in progressive contracture of eyelid tissue and distinction of PFH. These findings indirectly suggest that the risk of graft retraction is low.

Xeno-ADM has some limitations; it can only support the eyelid and without meibomis glandular secretion, the tear film may be destroyed, and ocular surface changes were not monitored in this study. Moreover, to combat scar contraction of the eyelid, eyelid margin adhesion is required for more than half a year, and a second operation is required; it is a long-duration, tiresome procedure, which may not be acceptable to all patients; it is a newer implant used in lid reconstruction which requires more cases and longer follow-up to rule out late complications.

## Conclusion

As a new biological material, the application of Xeno-ADM has gradually expanded with the gradual expansion of its nature. The eyelid blood supply is abundant, and Xeno-ADM easily survives and has good histocompatibility. After reconstruction, the eyelid has good cosmetic and functional outcomes, with few complications. The source was wide, and the acquisition was convenient. In conclusion, Xeno-ADM is a promising replacement for tarsal plates.

## Data Availability

The datasets generated during and analyzed during the current study are not publicly available due to human data but are available from the corresponding author on reasonable requestt.

## References

[1] Jank BJ, Goverman J, Guyette JP, et,al. Creation of a Bioengineered Skin Flap Scaffold with a Perfusable Vascular Pedicle. Tissue Eng Part A 2017 07;23 DOI:10.1089/ten.TEA.2016.0487. 10.1089/ten.tea.2016.0487 PMC5549829 28323545

[2] Hayek B, Hatef E, Nguyen M, Ho V, Hsu A, Esmaeli B. Acellular dermal graft (AlloDerm) for upper eyelid reconstruction after cancer removal. Ophthalmic Plast Reconstr Surg 2009;25(6) DOI:10.1097/IOP.0b013e3181b78989. 10.1097/IOP.0b013e3181b78989 19935241

[3] Actis AG, Actis G, De Sanctis U, Fea A, Rolle T, Grignolo FM. Eyelid benign and malignant tumors: issues in classification, excision and reconstruction. Minerva Chir. 2013;68 PMID:24172760. 24172760

[4] Mavrikakis I, Francis N, Poitelea C, Parkin B, Brittain P, Olver J. Medpor lower eyelid spacer: does it biointegrate? Orbit. 2009;28(1). 10.1080/01676830802414855. 10.1080/01676830802414855 19229747

[5] Ding J, Ma X, Xin Y, Li D. Correction of lower eyelid retraction with hard palate graft in the anophthalmic socket. Can J Ophthalmol. 2018;10;53(5). 10.1016/j.jcjo.2018.01.031. 10.1016/j.jcjo.2018.01.031 30340710

[6] Suga H, Ozaki M, Narita K, Kurita M, Shiraishi T, Ohura N, Takushima A, Harii K. Comparison of Nasal Septum and Ear Cartilage as a Graft for Lower Eyelid Reconstruction. J Craniofac Surg 2016 Mar;27(2), DOI:10.1097/SCS.0000000000002295. 10.1097/SCS.0000000000002295 26967067

[7] Krastinova D, Franchi G, Kelly MB (2002). Rehabilitation of the paralysed or lax lower eyelid using a graft of Conchal cartilage. Br J Plast Surg.

[8] Yim H, Cho YS, Seo CH, et al. The use of AlloDerm on major burn patients: AlloDerm prevents post-burn joint contracture. Burns. 2010;36(3). 10.1016/j.burns.2009.10.018. 10.1016/j.burns.2009.10.018 20080353

[9] Cho GW, Moon C, Song A (2021). Effect of growth factor-loaded acellular dermal matrix/MSCs on regeneration of chronic tympanic membrane perforations in rats. J Clin Med.

[10] Rohrich RJ, Reagan BJ, Adams WP Jr, Kenkel JM, Beran SJ. Early results of vermilion lip augmentation using acellular allogeneic dermis: an adjunct in facial rejuvenation. Plast Reconstr Surg 2000;105(01):409–416, discussion 417–418. DOI:10.1097/00006534-200001000-00065. 10.1097/00006534-200001000-00065 10627010

[11] Tork S, Jefferson RC, Janis JE. Acellular Dermal Matrices: Applications in Plastic Surgery. Semin Plast Surg. 2019;33(3). 10.1055/s-0039-1693019. 10.1055/s-0039-1693019 PMC6680075 31384233

[12] hridharani SM, Tufaro AP. A systematic review of acelluar dermal matrices in head and neck reconstruction. Plast Reconstr Surg 2012;130 DOI:10.1097/PRS.0b013e31825eff7a. 10.1097/PRS.0b013e31825eff7a 23096983

[13] Shorr N, Perry JD, Goldberg RA, Hoenig J, Shorr J (2000). The safety and applications of acellular human dermal allograft in ophthalmic plastic and reconstructive surgery: a preliminary report. Ophthal Plast Reconstr Surg.

[14] Chang M, Ahn SE, Baek S, The effect and applications of acellular dermal allograft (AlloDerm) in ophthalmic plastic surgery. J Craniomaxillofac Surg. 2014 Jul:42(5). 10.1016/j.jcms.2013.10.002. 10.1016/j.jcms.2013.10.002 24360752

[15] Ren B-C, Zhao J-Q, Zhang J (2007). Experimental study on eyelid reconstruction with acellular xenogenic dermal matrix. Zhonghua Yan Ke Za Zhi.

[16] Jing Li 1, Li Li Bai-Chao Ren, Experimental study of the eyelid reconstruction in situ with the acellular xenogeneic dermal matrix, Zhonghua Zheng Xing Wai Ke Za Zhi. 2007 23(2):154–7. PMID: 17554886. 17554886

[17] Ngo MD, Aberman HM, Hawes ML, Choi B, Gertzman AA. Evaluation of human acellular dermis versus porcine acellular dermis in an in vivo model for incisional hernia repair. Evaluation .Cell Tissue Bank 2011;12(2). DOI:10.1007/s10561-011-9245-5. 10.1007/s10561-011-9245-5 PMC3082045 21380733

[18] McGrath LA, Hardy TG, McNab AA Efficacy of porcine acellular dermal matrix in the management of lower eyelid retraction: case series and review of the literature. Graefes Arch Clin Exp Ophthalmol 2020;258(9). DOI:10.1007/s00417-020-04660-5. 10.1007/s00417-020-04660-5 32253504

[19] Adelman DM, Selber JC, Butler CE. Bovine versus Porcine Acellular Dermal Matrix: A Comparison of Mechanical Properties Plast Reconstr Surg Glob Open. 2014y:2(5). 10.1097/GOX.0000000000000072. 10.1097/GOX.0000000000000072 PMC4174084 25289348

[20] Ball JF, Sheena Y, TarekSaleh DM, Forouhi P, Benyon SL, Irwin MS, Malata CM. A direct comparison of porcine (Strattice™) and bovine (Surgimend™) acellular dermal matrices in implant-based immediate breast reconstruction. J Plast Reconstr Aesthet Surg 2017;70(8). DOI:10.1016/j.bjps.2017.05.015. 10.1016/j.bjps.2017.05.015 28624524

[21] Mazari FAK, Wattoo GM, Kazzazi NH, Kolar KM, Olubowale OO, Rogers CE, Azmy IA, TheComparisonof Strattice and SurgiMend in Acellular Dermal Matrix-Assisted, Implant-Based Immediate Breast Reconstruction.Plast Reconstr Surg 2018 02;141(2) DOI:10.1097/PRS.0000000000004018. 10.1097/PRS.0000000000004018 29369979

[22] Debels H, Hamdi M, Abberton K, Morrison W,Dermal matrices and bioengineered skin substitutes: a critical review of current options.Plast Reconstr Surg Glob Open 2015 ;3(1).DOI:10.1097/GOX.0000000000000219 10.1097/GOX.0000000000000219 PMC4323388 25674365

[23] DeGeorge BR, Ning B, Salopek LS (2017). Advanced imaging techniques for investigation of acellular dermal matrix biointegration. Plast Reconstr Surg.

[24] Zhuang A, Sun J, Zhang S, Zhou HF (2019). Acellular xenogenic dermal matrix as a spacer graft for treatment of eyelid retraction related to thyroid associated ophthalmopathy. Zhonghua Yan Ke Za Zhi..

[25] Barmettler A, Heo M. A Prospective, Randomized Comparison of Lower Eyelid Retraction Repair With Autologous Auricular Cartilage, Bovine Acellular Dermal Matrix (Surgimend), and Porcine Acellular Dermal Matrix (Enduragen) Spacer Grafts. Ophthalmic Plast Reconstr Surg 2018 ;34(3). DOI:10.1097/IOP.0000000000000946 10.1097/IOP.0000000000000946 28658181

